# Molineid nematodes of amphibians and reptiles: A checklist of Caribbean, Panamanian, and Neotropical species and notes on their biology and host associations

**DOI:** 10.1017/S0031182025101364

**Published:** 2026-02

**Authors:** Yuri Willkens, Jeannie Nascimento Santos, Francisco Tiago Vasconcelos Vasconcelos Melo

**Affiliations:** Laboratório de Biologia Celular e Helmintologia ‘Profª. Drª Reinalda Marisa Lanfredi’, Instituto de Ciências Biológicas, Universidade Federal do Pará, Belém, PA, Brazil.

**Keywords:** South America, Central America, herpetofauna, Nematoda, helminthology

## Abstract

Research on helminth parasites of amphibians and reptiles has a long-standing history and has seen continuous growth. Recent efforts by various authors to compile comprehensive checklists are crucial for advancing our understanding of parasite diversity, ecology and evolution. Nematodes belonging to the family Molineidae parasitize vertebrates worldwide, with the genera *Kentropyxia, Oswaldocruzia, Poekilostrongylus, Schulzia* and *Typhlopsia* identified as infecting amphibians and reptiles across the Neotropical and Panamanian regions. While these parasites are relatively common, there is a lack of updated identification keys and incomplete information about their morphology, biology, distribution and host range. In this paper, we conducted an extensive bibliographic survey of Molineidae nematodes in amphibians and reptiles and provide a checklist of 53 species found in the Neotropical and Panamanian regions, including the Caribbean islands, along with updated details on their diversity, host range and geographic distribution.

## Graphical abstract

**Figure:**
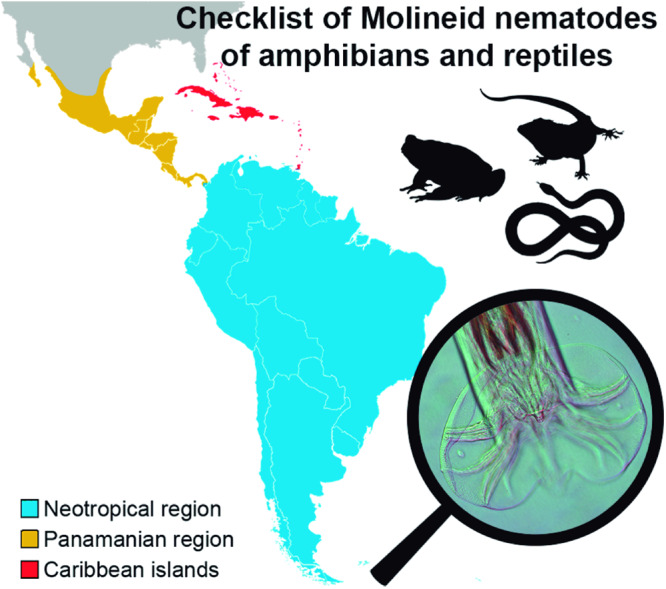

## Introduction

Helminth parasites of amphibians and reptiles have been studied for a long time, particularly from South American hosts. Research on this topic includes inventories dating back to Rudolphi (1819) and significant contributions from Travassos in the early 20th century. Additionally, species compilations were also presented by Baker (1987) and Vicente et al. (1993).

In recent years, research on helminth parasites of the herpetofauna has significantly increased. For instance, over the past decade, Ávila and Silva (2010), Campião et al. (2014), González and Hamann (2015a), Castillo et al. (2020) and Luque and Chero (2022) have made efforts to compile comprehensive checklists of helminths found in amphibians and reptiles. However, studies on helminth parasites still reveal gaps, including a lack of published manuscripts, local journals that disappeared, insufficiently detailed species descriptions and a scarcity of updated information sources (Laakso et al. 2021; Macedo et al. 2023). These challenges are particularly pronounced in studies cataloguing many species and/or those with poorly/insufficient morphological characters for the described species, with no clear diagnostic characters, leading to fragmented information and complicating data analysis.

Nematodes of the family Molineidae are known to parasitize the digestive tract of vertebrates worldwide. There are 9 genera of molineid nematodes that specifically infect amphibians and reptiles: *Schulzia* Travassos 1937; *Batrachostrongylus* Yuen 1937; *Poekilostrongylus* Schmidt and Whittaker 1975; *Trichoskrjabinia* Travassos 1937; *Typhlopsia* Baruš and Coy Otero 1978; *Kentropyxia* Baker 1982; *Bakeria* Moravec and Sey 1986; *Ragenema* Ben Slimane, Chabaud and Durette-Desset 1996; and *Oswaldocruzia* Travassos 1917 (Ben Slimane et al. 1996a). However, in neotropics, only species of the genera *Kentropyxia, Oswaldocruzia, Poekilostrongylus, Schulzia* and *Typhlopsia* have been reported (Ben Slimane et al. 1996a).

The genus *Schulzia* is characterized by a genital cone with small irregular digitations and by the absence of true cuticular ridges in the synlophe, with simple undulations instead. Males possess single-pointed, bifurcated or trifurcated spicules and caudal bursa with well-developed dorsal lobe, with rays 10 and 9 notably longer. Females possess a distinct ovojector, with a vestibule consisting of 3 muscular branches, the first being a prolongation of the vagina vera; the caudal spine is absent (Durette-Desset et al. 1985; Bursey et al. 2006a; González and Hamann, 2015b).

*Poekilostrongylus* is a monospecific genus, lacking both synlophe and alae. It has a long, thick dorsal ray forming a pointed dorsal lobe, and spicules divided into 3 branches: 1 long external-lateral and 2 short internal (Schmidt and Whittaker, 1975; Ben Slimane et al. 1996a). *Typhlopsia* is also a monospecific genus and also present a prominent pointed dorsal lobe but differs by the presence of synlophe with numerous crests (Baruš and Coy Otero, 1978).

In *Oswaldocruzia*, the *corona radiata* is absent and spicule morphology varies among biogeographical groups: (a) Oriento-Ethiopian, with 2–3 indistinct tips; (b) Neo-Ethiopian, with numerous indistinct tips; (c) Caribbean, with 3 main branches subdivided into numerous tips; and (d) Holarctic and (e) Neotropical, both with 3 branches forming a ‘fork’, ‘blade’ and ‘shoe’ (Ben Slimane et al. 1996a). The genus *Kentropyxia* is morphologically similar to *Oswaldocruzia*, but it can be readily distinguished by having a *corona radiata* encircling the oral aperture and unique 3-branched spicules: 1 pointed and 2 fan-like with numerous tips (Baker, 1982; Willkens et al. 2021, 2023).

To date, only a single identification key is available for nematode species within these genera (Ben Slimane et al. 1996a). This key covers only 1 of the 3 known species of *Kentropyxia* and 1 of the 4 described species of *Schulzia*. For *Oswaldocruzia*, the genus with the greatest number of described species, the key includes 74 of the 90 species currently recognized worldwide and only 29 of the 43 species known from the Neotropical and Panamanian realms. Also, recent studies on these genera have focused primarily on species descriptions, morphology and taxonomy. Information regarding their distribution and host range remains scattered and incomplete, molecular data are absent for most species, and their phylogenetic relationships are yet unknown.

This paper reviews the nematodes of the family Molineidae from amphibians and reptiles. We also provide a checklist of nematodes from the Caribbean, Panamanian and Neotropical regions. Additionally, we compile data about molineid nematodes’ diversity, host range, available molecular data and known geographic distribution.

## Materials and methods

### Literature research and review

We conducted a bibliographic survey using information from various scientific databases (BioOne, ISI, Jstor, PubMed, Scielo, Scopus and Web of Science) and published monographs. Incomplete records, data of unpublished theses or scientific meetings were not included.

Our review focused on all publications that referred to molineid parasites of amphibians and reptiles, specifically the genera *Kentropyxia, Oswaldocruzia, Poekilostrongylus, Schulzia* and *Typhlopsia* (along with their synonyms). We examine literature of molineid taxa from Neotropical and Panamanian regions published between 1819 and 2025. It included original species descriptions, checklists (Ávila and Silva, 2010; Campião et al. 2014; González and Hamman, 2015*a*), taxonomic keys (Ben Slimane et al. 1996a) and other publications with locality reports for all molineid taxa from amphibians and reptiles. Records not identified at species level were not included.

We also searched the published literature and the GenBank database for molecular data corresponding to each molineid taxon included in this study. Sequence data, when available, are listed by their accession numbers, including information regarding their respective hosts, localities and specific genomic region. Records not identified to species level were excluded.

### Taxonomy and terminology

The taxonomy of molineid species and terminology used here for anatomical structures follows Ben Slimane et al. (1996a), Anderson et al. (2009) and Gibbons (2010). The host taxonomy follows Frost (2025) and Uetz et al. (2025). Whenever the nomenclature of nematodes or hosts has changed and is not as in the original reference, the synonyms are given within parenthesis and discussed in the remarks. Some publications lack or do not specify information about hosts or localities; in these cases, it is also discussed in the remarks if the data is missing or given in posterior references.

We did not update the nomenclature for host species *Rhinella marina* (Linnaeus) and *Rhinella margaritifera* (Laurenti) groups which present cryptical diversity. Thus, for records in hosts of these species’ groups, we updated their nomenclature to *sensu lato*.

### Biogeographical division and localities

We followed the biogeographical division proposed by Holt et al. (2013) and used the Panamanian and Neotropical realms. However, since the Caribbean molineid species constitute a very particular morphological group (see Ben Slimane et al. 1996a), we present the Caribbean species as a separate group. Geographical records are given to the most specific level (municipalities and/or collection sites) according to the locations provided in the papers. When specific localities are absent, the geographical records are given only in the state/province/department or the country. The abbreviations for all localities cited in this paper are as follows:

Argentina (ARG): Astilleros (AST), Concepción del Bermejo (CON), Corrientes (COR), Ingeniero Juárez (INJ), Itaú River (ITR) and Jujuy Province (JUY), Las Lomitas (LOM) Taco Pozo (TAC).

Bahamas (BHS): Bahamian Field Station (BFS).

Bonaire (BES)

Brazil (BRA): Acre State (AC), Amazon State (AM) Alter do Chão (ALT), Angra dos Reis (ANG), Belém (BEL), Corcovado (CVD) Bocaina (BOC), Bodoquena (BOD), Botucatu (BOT), Cachimbó (CHB), Camisão Municipality (CAM), Carreiro Castanho (CCA), Caseara Municipality (CSR), Curitiba (CUR), Santa Teresa (SAN), Floresta Nacional de Caxiuanã (CAX), Gaviões (GAV), Ibitipoca State Park (IBT), Manaus State (MAN), Manguinhos (MGN), Marechal Floriano (MFL), Miguel Pereira Municipality (MGP), Novo Progresso Municipality (NVP), Parati (PRT), Petrópoles (PTR), Porto Walter (POW), Reserva Biológica de Sooretama (SOO), Reserva Extrativista Beija-Flor Brilho-de-Fogo (BBF), Rio Ituxi (ITX), Salobra (SLB), Miranda Municipality, Salvador (SVD), Salvaterra Municipality (SVT), Santa Candida Municipal Biological Reserve (SCM), Santarém (SNT), São Luís do Paraitinga (SLP), São Paulo City (SPC), São Sebastião Island (SSI), Serra do Mar State Park (SDM), Tijuca (TIJ), Três Barras Municipality (TRB), Vila Dois Rios (VDR) Ilha Grande, Volta Redonda (VTR), Rondônia State (RO) and Pernambuco State (PE), Petrolina (PET).

Chile (CHL): Fundo San Martin (FSM), La Picada (LPI), Mehuín (MEH), Puyehue (PUY).

Costa Rica (CRI): Área de Conservación Guanacaste (ACG), Cerro de La Muerte (CLM), Paso Ancho (PSA), San José (SJE), Tilarán (TIL), Heredia Province (HER), Punta Arenas Province (PUN), San José Province (SJP).

Cuba (CUB): Isla de la Juventud (JVT), Botany Garden (BTG), Cayo Guin (CGY), La Mariposa (MRP), La Vuelta (VLT), Las Cañas (CNS), Santiago de Cuba (STC).

Dominica (DMA): Saint Andrew Parish (SAP), Saint Patrick Parish (SPP) and Saint George Parish (SGP).

Dominican Republic (DOM): Baharona Province (BAH).

Ecuador (ECU): Hacienda Primavera (HPR), Reserva de Producción de Fauna Cuyabeno (RCU), San Lorenzo (SLZ), San Pablo (SPB), Santa Cecilia (SCE) and Sucumbiós (SUC).

French Guiana: Cayenne (CAY), Crique Grégoire (CRQ) and Paramana (PAR).

Guadeloupe (GLP).

Guyana (GUY): Rupununi District (RPD).

Haiti (HTI): Sud Department (SUD).

Mexico (MEX): Acapulco (ACA), Armeria (ARM), Benito Juárez (BJZ), Cerro del Tepezcuintle (CDT), Chairel (CHR), Champayán (CHM), Coquimatlán (COQ), El Carrizal (ELC), Huixtla (HXT), Julio Carrillo Ranch in Ticuizitán (JCR), Laguna Escondida in Los Tuxtlas (LGE), Las Palmas Road in Escuintla (LPR), Los Mogotes Laguna de Coyuca (LMO), Parque Estatal Lagunas de Yalahau (YLH), Rodolfo Figueroa Road (RFR), San Antonio (SAT), Teapa (TEA), Tres Palos (TRP), Vallarta-Las Palmas (VLP), Xtoloc (XTL).

Nicaragua (NIC): Isla Diamante (ISD).

Panama (PAN): Cerro Mali (CRM), El Aguacate (EAG), Panama City (PNC), San Blas Territory (SBT), Chiriqui Province (CHI), Canal Zone (CNZ).

Paraguay (PRY): Asunción (ASU), Chacoí (CHA), Estancia Estrellas (EES) and Remanso Castillo (RCA).

Peru (PER): Ucayali Region (UCA), Panguana (PNG), Parque Nacional Manu (PNM), Reserva Cuzco Amazónico (CUZ), San Martin (SMT), Yulitunqui (YUL), La Libertad Department (LBT), Ancash Department (ANC).

Puerto Rico (PRI): Cupey (CPY), Luquillo Forest (LUQ), El Yunque Mountain (YNQ).

Saint Vincent and the Grenadines (VCT): Soufriere Volcano (SFV).

Trinidad and Tobago (TTO): Saint Joseph (STJ).

Uruguay (URY): Montevideo (MTV).

Venezuela (VEN): Mérida (MER), Santa Rita (STR).

## Results

We recorded 53 species of molineid nematodes that parasitize amphibians and reptiles across the Panamanian, Caribbean and Neotropical regions. Among these, the genus *Oswaldocruzia* was the most represented, with 44 recorded species. This was followed by 4 species of *Schulzia*, 3 of *Kentropyxia*, 1 of *Poekilostrongylus* and 1 of *Typhlopsia*.

Regarding the hosts, we recorded molineids from 160 species across 31 families of amphibians and reptiles. Amphibians were the most numerous, comprising 146 species from 17 families. The anuran families recorded included Alsodidae (4 species), Batrachylidae (1 species), Brachycephalidae (1 species), Bufonidae (24 species), Ceratophryidae (1 species), Craugastoridae (5 species), Cycloramphidae (1 species), Dendrobatidae (1 species), Eleutherodactylidae (12 species), Hemiphractidae (1 species), Hylidae (17 species), Leptodactylidae (12 species), Microhylidae (1 species), Odontophrynidae (1 species), Ranidae (7 species) and Strabomantidae (8 species). In addition, the order Caudata was represented by a single family, Plethodontidae, which included 2 species (supplementary Table S1).

Reptiles were represented by 74 host species from 14 families. Among the lizards, we identified the following families and their respective number of species in each family: Alopoglossidae (3 species), Anolidae (28 species), Gekkonidae (1 species), Gymnophthalmidae (4 species), Iguanidae (2 species), Leiocephalidae (4 species), Leiosauridae (3 species), Phrynosomatidae (1 species), Scincidae (2 species), Teiidae (5 species), Tropidophiidae (1 species) and Tropiduridae (4 species). Additionally, we recorded 2 families of snakes: Colubridae (4 species) and Typhlopidae (1 species).

***List of species***

**Family Molineidae Durette-Desset and Chabaud, 1977**

**Genus *Kentropyxia* Baker, 1982**

***Kentropyxia bakeri* Willkens, Jesus, Borges, Ribeiro, Costa-Campos, Santos and Melo, 2023**

**Type hosts and type locality:** *Boana wavrini* (Parker) (CAX)

**Other hosts and locality records:** *Boana boans* (Linnaeus) (BBF), *Boana geographica* (Spix) (BBF)

**Host family:** Hylidae.

**Distribution:** Neotropical (Brazil).

**References:** Willkens et al. (2023).

***Kentropyxia hylae* Feitosa, Furtado, Santos and Melo, 2015**

**Type host and type locality:** *Osteocephalus taurinus* Steindachner (CAX)

**Host family:** Hylidae.

**Distribution:** Neotropical (Brazil).

**Available molecular data:** MK492922 from *O. taurinus* (CAX), partial COI sequence.

**References:** Feitosa et al. (2015); Willkens et al. (2021).

***Kentropyxia sauria* Baker, 1982**

**Type hosts and type locality:** *Kentropyx calcarata* Spix (BEL)

**Other hosts and locality records:** *Kentropyx calcarata* (NVP), *Kentropyx pelviceps* (Cope) (UCA), *Cnemidophorus gramivagus* McCrystal and Dixon (ALT)

**Host family:** Teiidae

**Distribution:** Neotropical (Brazil, Peru)

**References:** Baker (1982); Goldberg et al. (2007b); McAllister et al. (2010a); Goldberg et al. (2013).

**Genus *Oswaldocruzia* Travassos, 1917**

***Oswaldocruzia albareti* Ben Slimane and Durette-Desset, 1996**

**Type host and type locality:** *Rhinella marina* (= *Bufo marinus*) (CAY)

**Other hosts and locality records:** *Boana calcarata* (Troschel) (=*Hyla calcarata*) (SPB), *Boana fasciata* (Günther) (=*Hyla fasciata*) (SPB), *Boana geographica* (=*Hyla geographica*) (SPB), *Leptodactylus pentadactylus* (Laurenti) (CAY), *Rhinella margaritifera* (= *Bufo typhonius*) (CAY).

**Host families:** Bufonidae, Hylidae (Hylinae), Leptodactylidae.

**Distribution:** Neotropical (Ecuador and French Guyana).

**References:** Ben Slimane and Durette-Desset (1996a).

**Remarks:** For *O. albareti,* the authors incorrectly described this species with type II bursa. However, line drawings clearly show these nematodes with type III bursa. In the discussion section, Ben Slimane and Durette-Desset (1996a) also compare this species with congeners of type III bursa.

***Oswaldocruzia anolisi* Baruš and Coy Otero, 1968**

**Type hosts and type locality:** *Anolis equestris* Merrem (CGY)

**Other hosts and locality records:** *Anolis allisoni* Barbour (CU), *Anolis allogus* Barbour and Ramsden (CU), *Anolis baracoae* Schwartz (CU), *Anolis bartschi* (Cochran) (CU), *Anolis bremeri* Barbour (CU), *Anolis equestris* (CU)*, Anolis homolechis* (Cope) (CU), *Anolis loysiana* (Cocteau) (CU), *Anolis lucius* Duméril and Bibron (CU), *Anolis luteogularis* Noble and Hassler (CU), *Anolis porcus* (Cope) (=*Chamaleolis porcus*) (CU), *Anolis quadriocellifer* Barbour and Ramsden (CU), *Anolis sagrei* Duméril and Bibron (CU), *Caraiba andreae* (Reinhardt and Lütken) (=*Antillophis andreae*) (CU), *Cubophis cantherigerus* (Bibron) (=*Alsophis cantherigerus*) (CU), *Cyclura nubila* (Gray) (CU), *Leiocephalus carinatus* Gray (CU), *Leiocephalus cubensis* (Gray) (CU), *Leiocephalus macropus* (Cope) (CU), *Leiocephalus stictigaster* Schwartz (CU), *Pholidoscelis auberi* (Cocteau) (=*Ameiva auberi*) (CU), *Tropidophis pardalis* (Gundlach) (CU)

**Host family:** Anolidae.

**Distribution:** Caribbean (Cuba).

**References:** Baruš and Coy Otero (1968), Baruš and Coy Otero (1969), Coy Otero (1970), Baruš and Coy Otero (1978), Ben Slimane and Durette-Desset (1995b).

**Remarks**: In the studies conducted by Baruš and Coy Otero (1969) and Coy Otero (1970), *O. anolisi* was reported in many Iguanidae and Teiidae lizards from Cuba. In Baruš and Coy Otero (1978) this species was synonymized with *O. lenteixeirai*. However, Ben Slimane and Durette-Desset (1995c) re-examined the type-material and considered *O. anolisi* a valid species. Despite this, all posterior literature placed the reports from Baruš and Coy Otero (1969) and Coy Otero (1970) within *O. lenteixeirai*, despite recognizing *O. anolisi* as a separate species.

***Oswaldocruzia bainae* Ben Slimane and Durette-Desset, 1996**

**Type host and type locality:** *Anolis chrysolepis* Duméril and Bibron (SPB)

**Other hosts and locality records:** *Anolis biporcatus* (Wiegmann) (PNP), *Anolis fuscoauratus* D’orbigny (SPB), *Plica umbra* (Linnaeus) (SUC)

**Host families:** Anolidae, Tropiduridae

**Distribution:** Neotropical (Ecuador) and Panamanian (Panama)

**Reference:** Ben Slimane and Durette-Desset (1996b), Goldberg et al. (2009a), Bursey et al. (2003).

***Oswaldocruzia barusi* Ben Slimanei and Durette-Desset, 1995**

**Type hosts and type locality:** *Peltophryne empusa* Cope (=*Bufo empusus*) (CGY)

**Other hosts and locality records:** *Peltophryne dunni* (Barbour) (=*Bufo longinasus dunni*) (BTG, MRP, CNS, JVT, VLT), *Peltophryne empusa* (=*Bufo empusus*) (BTG, MRP, CNS, JVT, VLT), *Peltophryne fustiger* (Schwartz) (=*Bufo peltacephalus fustiger*) (BTG, MRP, CNS, JVT, VLT), *Peltophryne gundlachi* (Ruibal) (CUB), *Peltophryne taladai* (Schwartz) (=*Bufo taladai*) (BTG, MRP, CNS, JVT, VLT).

**Host family:** Bufonidae.

**Distribution:** Caribbean (Cuba).

**References:** Baruš (1973), Ben Slimane and Durette-Desset (1995c).

**Remarks:** Baruš (1973) identified parasites of various Bufonid host species from Cuba as *O. lenteixeirai*. However, these parasites were morphologically distinct, and Ben Slimane and Durette-Desset (1995c) allocated them to *O. barusi*.

***Oswaldocruzia belenensis* Santos, Giese, Maldonado Jr. and Lanfredi, 2008**

**Type host and type locality:** *Rhinella marina* (= *Chaunus marinus*) (BEL)

**Other hosts and locality records:** *Rhinella margaritifera* (CAX), *Rhinella marina* (SVT),

**Host family:** Bufonidae.

**Distribution:** Neotropical (Brazil).

**Available molecular data:** MK492915 from *R. margaritifera* (CAX), partial COI sequence; MK492916 from *R. marina* (BEL), partial COI sequence; MK492917 from *R. marina* (BEL), partial COI sequence; and MK492918 from *R. margaritifera* (CAX), partial COI sequence.

**References:** Santos et al. (2008), Willkens et al. (2021).

***Oswaldocruzia benslimanei* Durette-Desset, Alves dos Anjos and Vrcibradic, 2006**

**Type hosts and type locality:** *Enyalius bilineatus* (Duméril and Bibron) (MFL)

**Host family:** Leiosauridae

**Distribution:** Neotropical (Brazil)

**References:** Durette-Desset et al. (2006).

***Oswaldocruzia bonsi* Ben Slimane and Durette-Desset, 1993**

**Type host and type locality:** *Bolitoglossa equatoriana* Brame and Wake (SPB)

**Other hosts and locality records:** *Oreobates quixensis* Jiménez de la Espada (=*Ischnocnema quixensis*) (SPB)

**Host families:** Plethodontidae, Strabomantidae

**Distribution:** Neotropical (Ecuador)

**References:** Ben Slimane and Durette-Desset (1993).

***Oswaldocruzia brasiliensis* Lent and Freitas, 1935**

**Type hosts and type locality:** *Palusophis bifossatus* (Raddi) (= *Drymobius bifossatus, Dryadophis bifossatus, Mastigodryas bifossatus*) (MGN)

**Other hosts and locality records:** *Copeoglossum nigropunctatum* (Spix) (=*Mabuya nigropunctata*) (UCA), *Erythrolamprus miliaris* (Linnaeus) (=*Liophis miliaris*) (MGN), *Hemidactylus mabouia* (Moreau de Jonnès) (RJ)

**Host family:** Colubridae (Colubrinae, Dipsadinae)

**Distribution:** Neotropical (Brazil, Peru)

**References:** Lent and Freitas (1935), Freitas (1956), Rodrigues and Santos (1974), Vicente et al. (1993) and McAllister et al. (2010a).

***Oswaldocruzia brevispicula* Moravec and Kaiser, 1995**

**Type hosts and type locality:** *Pristimantis shrevei* (Schwartz) (= *Eleutherodactylus shrevei*) (SFV)

**Host family:** Strabomantidae

**Distribution:** Caribbean (Saint Vincent and the Grenadines)

**References:** Moravec and Kaiser (1995)

***Oswaldocruzia burseyi* Durette-Desset, Alves dos Anjos and Vrcibradic, 2006**

**Type hosts and type locality:** *Enyalius perditus* Jackson (SSI)

**Other hosts and locality records:** *Enyalius perditus* (SCM)

**Host family:** Leiosauridae

**Distribution:** Neotropical (Brazil)

**References:** Durette-Desset et al. (2006), Vrcibradic et al. (2008), Barreto-Lima et al. (2012)

***Oswaldocruzia cartagoensis* Bursey and Goldberg, 2011**

**Type hosts and type locality:** *Bolitoglossa subpalmata* (Boulenger) (CLM)

**Host family:** Plethodontidae

**Distribution:** Panamanian (Costa Rica)

**References:** Bursey and Goldberg (2011)

***Oswaldocruzia cassonei* Ben Slimane and Durette-Desset, 1996**

**Type host and type locality:** *Pristimantis lanthanites* (Lynch) (=*Eleutherodactylus lanthanites*) (SPB)

**Other hosts and locality records:** *Pristimantis altamazonicus* (Barbour and Dunn) (=*Eleutherodactylus altamazonicus*) (SPB), *Pristimantis conspicillatus* (Günther) (=*Eleutherodactylus conspicillatus*) (SPB), *Pristimantis diadematus* (Jiménez de la Espada) (=*Eleutherodactylus diadematus*) (SPB)

**Host family:** Strabomantidae

**Distribution:** Neotropical (Ecuador)

**Reference:** Ben Slimane and Durette-Desset (1996b)

***Oswaldocruzia chabaudi* Ben Slimane and Durette-Desset, 1996**

**Type host and type locality:** *Boana boans* (=*Hyla boans*) (SPB)

**Other hosts and locality records:** *Boana fasciata* (=*Hyla fasciata*) (SPB), *Boana geographica* (=*Hyla geographica*) (SPB, CAX), *Boana wavrini* (CAX), *Itapotihyla langsdorffiii* (Duméril & Bibron) (SAN). *Osteocephalus cabrerai* (BBF)

**Host family:** Hylidae (Hylinae)

**Distribution:** Neotropical (Ecuador, Brazil)

**Available molecular data:** MK492919 from *Boana wavrini* (CAX), partial COI sequence; and MK492920 from *B. geographica* (CAX), partial COI sequence.

**References:** Ben Slimane and Durette-Desset (1996a), Willkens et al. (2021), Neves et al. (2024), Vrcibradic et al. (2025).

***Oswaldocruzia chambrieri* Ben Slimane and Durette-Desset, 1993**

**Type host and type locality:** *Rhinella margaritifera* (=*Bufo typhonius*) (SPB)

**Other hosts and locality records:** *Amazophrynella bokermanni* (Izecksohn) (CAX), *Rhinella margaritifera* (CAX, HPR)

**Host family:** Bufonidae

**Distribution:** Neotropical (Ecuador, Brazil)

**Available molecular data:** MK492921 from *A. bokermanni* (CAX), partial COI sequence; and KU980934 from *R. margaritifera* (CAX), partial COI sequence.

**References:** Ben Slimane and Durette-Desset (1993), Willkens et al. (2016), Willkens et al. (2021)

***Oswaldocruzia costaricensis* Bursey and Goldberg, 2005**

**Type hosts and type locality:** *Lithobates* cf. *forreri* (Boulenger) (=*Rana* cf. *forreri*) (GUA)

**Other hosts and locality records:** *Agalychnis callidryas* (Cope) (CR), *Anolis aquaticus* (Taylor) (ACG), *Anolis lionotus* Cope (ACG), *Craugastor fitzingeri* (Schmidt) (CR, ACG), *Craugastor gollmeri* (Peters) (CR), *Craugastor ranoides* (Cope) (CR), *Craugastor taurus* (Taylor) (CR), *Ctenosaura quinquecarinata* (Gray) (ACG), *Incilius coccifer* (Cope) (ACG), *Incilius luetkenii* (Boulenger) (ACG), *Lithobates taylori* (Smith) (CR), *Lithobates warszewitschii* (Schmidt) (GUA, PUN, SJP), *Rhaebo haematiticus* (Cope) (ACG), *Rhinella marina* (ACG), *Sceloporus variabilis* Wiegmann (ACG), *Scinax elaeochroa* (Cope) (CR), *Smilisca phaeota* (Cope) (CR).

**Host family:** Anolidae, Bufonidae, Craugastoridae, Hylidae, Iguanidae, Phrynosomatidae, Ranidae.

**Distribution:** Panamanian (Costa Rica)

**References:** Bursey and Goldberg (2005), Bursey and Goldberg, (2007), Bursey et al. (2007a), Goldberg and Bursey (2007a), Goldberg and Bursey (2008a), Goldberg and Bursey (2008b), Bursey (2010), Bursey and Brooks (2010)

***Oswaldocruzia dlouhyi* Ben Slimane and Durette-Desset 1995**

**Type hosts and type locality:** *Rhinella* sp. (=*Bufo* sp.) (GAV)

**Host family:** Bufonidae

**Distribution:** Neotropical (Brazil)

**References:** Ben Slimane and Durette-Desset 1995*b*

***Oswaldocruzia dorsarmata* Ben Slimane, Durette-Desset and Chabaud, 1995**

**Type hosts and type locality:** *Anolis marmoratus* Duméril and Bibron (GLP)

**Host family:** Anolidae

**Distribution:** Caribbean (Guadeloupe)

**References:** Ben Slimane et al. (1995a)

***Oswaldocruzia franciscoensis* Vieira, Pereira, Ribeiro, Oliveira, Silva, Muniz-Pereira and Felix-Nascimento, 2023**

**Type hosts and type locality:** *Leptodactylus macrosternum* Miranda-Ribeiro (PET)

**Host family:** Leptodactylidae

**Distribution:** Neotropical (Brazil)

**References:** Vieira et al. (2023)

***Oswaldocruzia fredi* Durette-Desset, Alves dos Anjos and Vrcibradic, 2006**

**Type hosts and type locality:** *Enyalius iheringii* Boulenger (SSI)

**Host family:** Leiosauridae

**Distribution:** Neotropical (Brazil)

**References:** Durette-Desset et al. (2006); Vrcibradic et al. (2008),

***Oswaldocruzia jeanbarti* Ben Slimane, Durette-Desset and Chabaud, 1995**

**Type hosts and type locality:** *Anolis marmoratus* (GLP)

**Host family:** Anolidae

**Distribution:** Caribbean (Guadeloupe)

**References:** Ben Slimane et al. (1995a)

***Oswaldocruzia lamotheargumedoi* Ruiz-Torres, García-Prieto, Osorio-Sarabia and Violante-González, 2013**

**Type hosts and type locality:** *Rhinella marina* (LMO)

**Host family:** Bufonidae

**Distribution:** Panamanian (Mexico)

**References:** Ruiz-Torres et al. (2013)

***Oswaldocruzia lanfrediae* Larrat, Melo, Gomes, Willkens and Santos, 2018**

**Type hosts and type locality:** *Leptodactylus paraensis* Heyer (CAX)

**Host family:** Leptodactylidae

**Distribution:** Neotropical (Brazil)

**References:** Larrat et al. (2018)

***Oswaldocruzia lenteixeirai* Vigueras, 1938**

**Type hosts and type locality:** *Osteopilus septentrionalis* (Duméril and Bibron) (=*Hyla insulsa, Hyla septentrionalis*) (CUB)

**Other hosts and locality records:** *Anolis armouri* (Cochran) (SUD), *Anolis bahorucoensis* Noble and Hassler (BAH), *Anolis bonairensis* Ruthven (BES), *Aquarana catesbeiana* Shaw (=*Rana catesbeiana*) (CUB), *Eleutherodactylus atkinsi* Dunn (CUB), *Eleutherodactylus coqui* Thomas (LUQ), *Eleutherodactylus cuneatus* (Cope) (= *Eleutherodactylus sierramaestrae*) (CUB), *Eleutherodactylus dimidiatus* (Cope) (CUB), *Eleutherodactylus goini* Schwartz (CUB), *Eleutherodactylus greyi* Dunn (CUB), *Eleutherodactylus klinikowskii* Schwartz (CUB), *Eleutherodactylus pinarensis* Dunn (CUB), *Eleutherodactylus planirostris* (Cope) (CUB), *Eleutherodactylus portoricensis* Schmidt (CPY), *Eleutherodactylus zeus* Schwartz (CUB), *Eleutherodactylus zugi* Schwartz (CUB), *Osteopilus septentrionalis* (Duméril and Bibron, 1841) (BFS)

**Host families:** Hylidae (Hylinae), Anolidae, Ranidae, Eleutherodactylidae,

**Distribution:** Caribbean (Bahamas, Bonaire, Cuba, Dominican Republic, Haiti, Puerto Rico)

**References:** Vigueras (1938), Baruš and Moravec (1967), Baruš and Coy Otero (1968), Baruš (1972), Baruš (1973), Schmidt and Whittaker (1975), Baruš and Coy Otero (1978), Coy Otero and Baruš (1979), Martinez et al. (1982), Coy Otero and Ventosa (1984), Goldberg et al. (1994), Ben Slimane and Durette-Desset (1995c), Moravec and Kaiser (1995), Goldberg et al. (1996), Goldberg et al. (1998), Goldberg and Bursey (2019)

**Remarks:** Prokopic (1957) reported *O. lenteixeirai* in *Rana dalmatina* Fitzinger from the former Czechoslovakia; however, Moravec and Vojtková (1975) later identified this as misidentification. Baruš and Moravec (1967) redescribed *O. lenteixeirai* using specimens from the type host and type locality, but these were subsequently transferred to *O. moraveci* by Ben Slimane and Durette-Desset (1995c). Additionally, *O. lenteixeirai* identified by Baruš (1973) in various bufonid host species from Cuba were assigned by Ben Slimane and Durette-Desset (1995c) to *O. barusi*. Similarly, *O. lenteixeirai* identified by Goldberg and Bursey (1996) were redefined to *O. marechali* by Goldberg et al. (1997).

Baruš and Coy Otero (1978) synonymized *O. anolisi* with *O. lenteixeirai*, a classification later considered inaccurate by Ben Slimane and Durette-Desset (1995c). However, all subsequent literature continued to consider the reports of *O. anolisi* from Baruš and Coy Otero (1969) and Coy Otero (1970) in numerous Iguanidae and Teiidae lizards in Cuba as belonging to *O. lenteixeirai*, even while they treated as separate species with a distinct diagnosis. Thus, we have decided to categorize all reports of nematodes previously identified as *O. anolisi* separately from this species.

***Oswaldocruzia lescurei* Ben Slimane and Durette-Desset, 1996**

**Type host and type locality:** *Rhinella margaritifera* (= *Bufo typhonius*) (PAR)

**Other hosts and locality records:** *Incilius marmoreus* (Wiegmann) (CDT), *Rhinella margaritifera* (= *Bufo typhonius*) (PAR, CRQ)

**Host family:** Bufonidae

**Distribution:** Neotropical (French Guyana) and Panamanian (Mexico)

**References:** Ben Slimane and Durette-Desset (1996a), Trejo-Meléndez et al. (2019).

**Remarks:** For several years, the definition of *Rana typhonia* was unclear, leading to confusion with other species names, such as *Bufo typhonius*, which has been associated with different amphibians from the families Bufonidae and Hylidae. Trejo-Meléndez et al. (2019) consider *Bufo typhonius* from Ben Slimane and Durette-Desset (1996a) as a synonym of *Trachycephalus typhonius* (Hylidae). However, Ben Slimane and Durette-Desset (1996a) treated these hosts as bufonids, not hylids. Therefore, we have decided to update this classification to *R. margaritifera.*

***Oswaldocruzia lopesi* Freitas and Lent, 1938**

**Type hosts and type locality:** *Leptodactylus latrans* (Steffen) (=*Leptodactylus ocellatus*) (MGN)

**Other hosts and locality records:** *Ameerega picta* (Tschudi) (=*Epipedobates pictus*) (CUZ), *Boana fasciata* (=*Hyla fasciata*) (CUZ), *Hamptophryne boliviana* (Parker) (CUZ), *Leptodactylus bolivianus* Boulenger (CUZ), *Leptodactylus latrans* (=*Leptodactylus ocellatus*) (CAM, SLB, SOO, MTV), *Pristimantis fenestratus* (Steindachner) (CUZ), *Rhaebo glaberrimus* (Günther) (CUZ), *Rhinella icterica* (Spix) (=*Bufo ictericus*) (MGP), *Rhinella margaritifera* (CUZ), *Rhinella marina* (=*Bufo marinus*) (MAN), *Trachycephalus coriaceus* (Peters) (=*Phrynohyas coriacea*) (CUZ)

**Host family:** Bufonidae

**Distribution:** Neotropical (Brazil, Peru, Uruguay)

**References:** Freitas and Lent (1938), Travassos et al. (1939), Freitas (1956), Bursey et al. (2001), Gonçalves et al. (2002), Luque et al. (2005)

***Oswaldocruzia manuensis* Guerrero, 2013**

**Type hosts and type locality:** *Rhinella marina* (PNM)

**Host family:** Bufonidae

**Distribution:** Neotropical (Peru)

**References:** Guerrero (2013)

***Oswaldocruzia marechali* Ben Slimane, Durette-Desset and Chabaud, 1995**

**Type hosts and type locality:** *Anolis marmoratus* (GLP)

**Other hosts and locality records:** *Anolis oculatus* (Cope) (SAP, SPP, SGP)

**Host family:** Anolidae

**Distribution:** Caribbean (Dominica and Guadeloupe)

**References:** Ben Slimane et al. (1995a), Goldberg and Bursey (1996), Goldberg et al. (1997)

**Remarks:** This was previously identified as *O. lenteixeirai* by Goldberg and Bursey (1996) as redefined by Goldberg et al. (1997).

***Oswaldocruzia mauleoni* Ben Slimane, Durette-Desset and Chabaud, 1995**

**Type hosts and type locality:** *Anolis marmoratus* (GLP)

**Host family:** Anolidae

**Distribution:** Caribbean (Guadeloupe)

**References:** Ben Slimane et al. (1995a)

***Oswaldocruzia mazzai* Travassos, 1935**

**Type host and type locality:** *Rhinella diptycha* (= *Bufo marinus*) (JUY)

**Other hosts and locality records:** *Anolis brasiliensis* Vanzolini and Williams (=*Anolis nitens*) (RPD), *Boana raniceps* (Cope) (=*Hypsiboas raniceps, Hyla spegazzini*), *Leptodactylus bufonius* Boulenger (SLB, BOD), *Leptodactylus fuscus* (Schneider) (CSR), *Leptodactylus latrans* (= *Leptodactylus ocellatus, Cystignathus ocellatus*) (CSR), *Leptodactylus mystaceus* (Spix) (SCE), *Leptodactylus pentadactylus* (SCE), *Leptodactylus pustulatus* (Peters) (CSR), *Pristimantis altamazonicus* (=*Eleutherodactylus altamazonicus*) (SCE), *Rhinella diptycha* (Cope) (=*Bufo marinus, Rhinella schneideri, Bufo paracnemis*) (SLB, BOD), *Rhinella icterica* (=*Bufo ictericus*) (MGP), *Rhinella margaritifera* (=*Bufo typhonius*) (SCE, MAN, RPD), *Rhinella marina* (=*Bufo marinus*) (SPB, MAN, RPD), *Rhinella major* (TAC), *Tropidurus torquatus* (Wied-Neuwied) (CHB, BOD).

**Host families:** Anolidae, Bufonidae, Hylidae (Hylinae), Leptodactylidae, Strabomantidae, Tropiduridae.

**Distribution:** Neotropical (Argentina, Brazil, Ecuador, Guyana)

**Reference:** Travassos (1935); Travassos (1937); Travassos et al. (1939); Lent et al. (1946); Freitas (1956); Masi-Pallares and Maciel (1974); Dyer and Altig (1977); Vicente et al. (1991); Vicente et al. (1993); Ben Slimane and Durette-Desset (1995a), Ben Slimane and Durette-Desset (1995b); Gonçalves et al. (2002); Luque et al. (2005); Goldberg et al. (2009b); McAllister et al. (2010b), Hamann and González (2015)

**Remarks:** This species was first described by Travassos (1935) in *Bufo* sp., which was later updated to *R. marina* (=*Bufo marinus*) in Travassos et al. (1939). However, since this species does not occur in Argentina, we recognize *R. diptycha*, a member of the *R. marina* group that has known distribution in Argentina, as the type host.

The specimens reported by Lent et al. (1946) in amphibians from Paraguay were later identified as *O. proencai* Ben Slimane and Durette-Desset (1995b) and were not considered here. Gonçalves et al. (2002) argued that Freitas (1956) incorrectly referred to *B. parachinemis* as a synonym of *B. marinus*. However, it did not seem to us that Freitas (1956) considers both names as synonyms; instead, he highlights the correction given by Travassos and Freitas (1942) of the host’s name presented in Travassos (1937). Freitas (1956) also synonymized *O. subauricularis* sensu Travassos et al. (1939) to *O. mazzai*.

***Oswaldocruzia moraveci* Ben Slimanei and Durette-Desset, 1995**

**Type hosts and type locality:** *Osteopilus septentrionalis* (*=Hyla insula*) (CGY)

**Host family:** Bufonidae

**Distribution:** Caribbean (Cuba)

**References:** Baruš and Coy Otero (1968), Ben Slimane and Durette-Desset (1995c)

**Remarks**: Baruš and Moravec (1968) reported specimens of *O. lenteixeirai* from the same host and locality of the type material. Ben Slimane and Durette-Desset (1995c) re-examined these specimens and pointed out several characters that justified the proposal of *O. moraveci* (= *O. lenteixeirai* sensu Baruš and Moravec, 1967, nec; Vigueras, 1938).

***Oswaldocruzia neghmei* Puga, 1981**

**Type hosts and type locality:** *Hylorina sylvatica* Bell (PUY)

**Other hosts and locality records:** *Alsodes vittatus* (Philippi) (=*Eupsophus vittatus*) (FSM, MEH, LPI), *Eupsophus migueli* Formas (MEH), *Eupsophus roseus* (Duméril and Bibron) (FSM), *Eupsophus vertebralis* Grandison (FSM, MEH)

**Host family:** Batrachylidae, Alsodidae

**Distribution:** Neotropical (Chile)

**References:** Puga (1980), Puga (1981), Puga (1994)

***Oswaldocruzia nicaraguensis* Bursey, Goldberg and Vitt, 2006**

**Type hosts and type locality:** *Holcosus festivus* (Lichtenstein and Martens) (=*Ameiva festiva*) (ISD)

**Other hosts and locality records:** *Anolis biporcatus* (PUN, EAG), *Anolis capito* Peters (=*Norops capito*) (RSJ, PNP), *Anolis humilis* Peters (ISD, HER, CHI), *Anolis limifrons* Cope (ISD, HER, CNZ, SBT), *Anolis lionotus* (ISD, PNP), *Scincella cherriei* (Cope) (=*Sphenomorphus cherriei*) (CR)

**Host family:** Teiidae, Scincidae, Anolidae

**Distribution:** Panamanian (Nicaragua, Costa Rica, Panama)

**References:** Bursey et al. (2006b), Bursey et al. (2007b), Bursey et al. (2007a), Goldberg et al. (2010), Bursey et al. (2012)

***Oswaldocruzia panamaensis* Bursey, Goldberg and Telford Jr. 2007**

**Type hosts and type locality:** *Loxopholis rugiceps* Cope (=*Leposoma rugiceps*) (PNC)

**Host family:** Gymnophthalmidae

**Distribution:** Panamanian (Panama)

**References:** Bursey et al. (2007a)

***Oswaldocruzia peruensis* Ben Slimane, Verhaagh and Durette-Desset, 1995**

**Type hosts and type locality:** *Stenocercus roseiventris* D’orbigny In Duméril and Bibron (PNG)

**Other hosts and locality records:** *Anolis punctatus* Daudin (CUZ), *Stenocercus roseiventris* (CUZ)

**Host families:** Tropiduridae, Anolidae

**Distribution:** Neotropical (Peru)

**References:** Ben Slimane et al. (1995b), Bursey et al. (2005)

***Oswaldocruzia petterae* Ben Slimane and Durette-Desset, 1996**

**Type host and type locality:** *Leptodactylus pentadactylus* (SPB)

**Host family:** Leptodactylidae

**Distribution:** Neotropical (Ecuador)

**Reference:** Ben Slimane and Durette-Desset (1996b)

***Oswaldocruzia proencai* Ben Slimane and Durette-Desset, 1995**

**Type host and type locality:** *Rhinella diptycha* (=*Bufo paracnemis, Rhinella schneideri*) (PRY)

**Other hosts and locality records:** *Gastrotheca peruana* (Boulenger) (LBT, ANC), *Leptodactylus bufonius* (ASU, CHA, RCA), *Leptodactylus fuscus* (CSR), *Leptodactylus latrans* (= *Leptodactylus ocellatus*) (ASU, CHA, RCA), *Leptodactylus pustulatus* (CSR), *Rhinella arenarum* (Hensel) (= *Chaunus arenarum*) (AST, ITR), *Rhinella diptycha* (=*Rhinella schneideri*) (ASU, CHA, RCA, ITR, COR), *Rhinella margaritifera* (=*Bufo typhonius*) (UCA)

**Host families:** Bufonidae, Hemiphractidae, Leptodactylidae

**Distribution:** Neotropical (Argentina, Brazil, Paraguay, Peru)

**References:** Lent et al. (1946); Vicente et al. (1991), Ben Slimane and Durette-Desset (1995b), Ramallo et al. (2007a), Ramallo et al. (2007b), González and Hamann (2008), Goldberg et al. (2009b) and McAllister et al. (2010a), Gómez et al. (2020)

**Remarks:** Lent et al. (1946) reported specimens of *O. mazzai* in *Rhinella diptycha* (=*Bufo paracnemis*), *Leptodactylus latrans* (= *Leptodactylus ocellatus*) and *L. bufonius* from Paraguay. However, Ben Slimane and Durette-Desset (1995b), after a further examination of those specimens, proposed a new species, reassigning those nematodes to *O. proencai*.

***Oswaldocruzia subauricularis* (**Rudolphi, 1819**) Travassos, 1917 (=*Strongylus subauricularis*)**

**Type host and type locality:** *Rhinella ornata* (Spix) (= *Rana musica, Bufo musicus, Bufo lentiginosus, Bufo ornatus*) (BR)

**Other hosts and locality records:** *Boana faber* (Wied-Neuwied) (=*Hyla faber*) (ANG), *Ceratophrys cornuta* (Linnaeus) (PTP), *Enyalius perditus* (IBT), *Incilius marmoreus* (CDT), *Leptodactylus latrans* (*= Leptodactylus ocellatus, Cystignathus ocellatus*) (VTR, SVD, SLP), *Leptodactylus melanonotus* (Hallowell) (COQ, ARM, TRP, ACA, ELC, VLP, SAT, BJZ, TEA, CHM, CHR), *Leptodactylus pentadactylus* (SVD), *Lithobates brownorum* (Sanders) (YLH), *Lithobates* cf. *forreri* (=*Rana* cf. *forreri*) (ACA, RFR, LPR), *Lithobates* sp. (JCR), *Lithobates vaillanti* (Brocchi) (=*Rana vaillanti*) (LGE), *Phyllomedusa burmeisteri* Boulenger (ANG), *Physalaemus olfersii* (Lichtenstein and Martens) (SDM), *Rhinella crucifer* (Wied-Neuwied) (=*Bufo crucifer*) (ANG), *Rhinella diptycha* (=*Bufo paracnemis*) (SVD, INJ), *Rhinella dorbignyi* (Duméril and Bibron) (=*Rhinella fernadezae*) (COR), *Rhinella horribilis* (Wiegmann) (=*Bufo horribilis*) (HXT), *Rhinella icterica* (=*Bufo marinus bimaculatus*) (CUR, MGP, BOT), *Rhinella marina* (=*Bufo agua, Bufo marinus*) (PRT, BOC, SPC, SLB, PSA, SJE, TIL, XTL), *Rhinella ornata* (=*Bufo ornatus*) (BR), *Rhinella* sp. (=*Bufo* sp.) (GAV), *Trachycephalus mesophaeus* (Hensel) (*= Hyla mesophaea, Phrynohias mesophaea*) (ANG)

**Host families:** Bufonidae, Ceratophryidae, Hylidae (Hylinae, Phylomedusinae), Leiosauridae, Leptodactylidae, Ranidae and Strabomantidae

**Distribution:** Neotropical (Argentina, Brazil) and Panamanian (Costa Rica, Mexico)

**Remarks:** Rudolphi (1819) first described this species from the host ‘*Rana musica*’ in Brazil. This host name was later updated to *Bufo musicus* by Travassos (1917). Travassos (1921) states that *B. musicus* corresponded to *B. lentiginosus americanus* collected by Natterer from Brazil.

Ben Slimane and Durette-Desset (1993) recommend following the redescription provided by Travassos (1937), although they noted that the host could be either *Bufo aguae* (a name previously used for *R. marina, R. icterica* and *R. crucifer*) or *Ceratophrys cornuta*. According to Ben Slimane and Durette-Desset (1995b) the original host determination was likely incorrect, as the name was synonymous with *Bufo americanus* and *Bufo terrestris* (both of which are currently assigned to genus *Anaxyrus*). These species are native to North America and do not occur in Brazil.

The name *B. lentiginosus* has also been used for several species of Bufonidae across North America and South America, but only *Rhinella ornata* is found in Brazil and was documented with the original material. Thus, we opted to update this name to *R. ornata*.

Freitas (1955) reported *O. subauricularis* (Rudolphi, 1819) in *Enyalius perditus* (=*Enyalius catenatus*) from Tijuca, Rio de Janeiro, Brazil. These nematode specimens were considered *species inquirenda* by Durette-Desset et al. (2006).

**References:** Rudolphi (1819), Travassos (1917, 1921), Pearse (1936), Travassos (1937), Caballero (1949), Fahel (1952), Freitas (1956), Brenes-Madrigal and Bravo-Hollis (1959), Tantalean (1976), Vicente and Santos (1976), Rodrigues et al. (1982), Baker (1987), Vicente et al. (1991), Ben Slimane and Durette-Desset (1995a), Ben Slimane and Durette-Desset (1995b), Paredes-Calderón et al. (2004), Luque et al. (2005), Cabrera-Guzmán et al. (2007), Espinoza-Jiménez et al. (2007), Sousa et al. (2007), Pinhão et al. (2009), Cabrera-Guzmán et al. (2010), Yáñez-Arenas and Guillén-Hernández (2010), Mata-López et al. (2013), Toledo et al. (2013), Toledo et al. (2015), Hamann et al. (2013), Velázquez-Urrieta and León-Règagnon (2018), Trejo-Meléndez et al. (2019), González et al. (2021).

***Oswaldocruzia taranchoni* Ben Slimane and Durette-Desset 1995**

**Type hosts and type locality:** *Rhinella marina* (=*Bufo marinus*) (PE)

**Host family:** Bufonidae

**Distribution:** Neotropical (Brazil)

**References:** Ben Slimane and Durette-Desset 1995*b*

***Oswaldocruzia tcheprakovae* Ben Slimane and Durette-Desset, 1996**

**Type host and type locality:** *Pristimantis altamazonicus* (=*Eleutherodactylus altamazonicus*) (SPB)

**Host family:** Strabomantidae

**Distribution:** Neotropical (Ecuador)

**Reference:** Ben Slimane and Durette-Desset (1996b)

***Oswaldocruzia touzeti* Ben Slimane and Durette-Desset, 1993**

**Type host and type locality:** *Pristimantis variabilis* (Lynch) (=*Eleutherodactylus variabilis*) (SPB)

**Host family:** Strabomantidae

**Distribution:** Neotropical (Ecuador)

**References:** Ben Slimane and Durette-Desset (1993)

***Oswaldocruzia urubambaensis* Guerrero, 2013**

**Type hosts and type locality:** *Rhinella marina* (SMT)

**Host family:** Bufonidae

**Distribution:** Neotropical (Peru)

**References:** Guerrero (2013)

***Oswaldocruzia vaucheri* Ben Slimane and Durette-Desset, 1993**

**Type host and type locality:** *Oreobates quixensis* (=*Ischnocnema quixensis*) (SPB)

**Other hosts and locality records:** *Leptodactylus fuscus* (NVP)

**Host families:** Strabomantidae, Leptodactylidae

**Distribution:** Neotropical (Ecuador, Brazil)

**References:** Ben Slimane and Durette-Desset (1993); Goldberg et al. (2007b)

***Oswaldocruzia venezuelensis* Ben Slimane, Guerrero and Durette-Desset, 1996**

**Type hosts and type locality:** *Rhinella marina* (=*Bufo marinus*) (STR)

**Other hosts and locality records:** *Rhinella marina* (=*Bufo marinus*) (STJ)

**Host family:** Bufonidae

**Distribution:** Neotropical (Venezuela, Trinidad and Tobago)

**References:** Ben Slimane et al. (1996b), Ragoo and Omah-Maharaj (2003)

***Oswaldocruzia vitti* Bursey and Goldberg, 2004**

**Type-host and type-locality:** *Cercosaura eigenmanni* (Griffin) (=*Prionodactylus eigenmanni*) (AM)

**Other hosts and locality records:** *Alopoglossus angulatus* (Linnaeus) (AC, RO, SUC), *Alopoglossus atriventris* Duellman (AC), *Anolis fuscoauratus* (SNT, POW, ITX, CCA, SUC, RCU), *Anolis punctatus* (AM, RO, SUC, RCU), *Cercosaura eigenmanni* (=*Prionodactylus eigenmanni*) (RO, CUZ), *Cercosaura ocellata* Wagler (GUA), *Cercosaura oshaughnessyi* (Boulenger) (=*Prionodactylus oshaughnessyi*) (AC, SUC, RCU), *Cnemidophorus gramivagus* (ALT), *Plica plica* (Linnaeus) (AC, PA, RO), *Plica umbra* (PA, RO)

**Host families:** Alopoglossidae, Anolidae, Gymnophthalmidae, Teiidae, Tropiduridae

**Distribution:** Neotropical (Brazil, Ecuador, Peru)

**References:** Bursey and Goldberg (2004); Bursey et al. (2005); Goldberg et al. (2006a); Goldberg et al. (2006b); Goldberg et al. (2007a), Goldberg et al. (2009a), Ávila and Silva (2011), Goldberg et al. (2013).

**Genus *Poekilostrongylus* Schmidt and Whittaker, 1975**

***Poekilostrongylus puertoricensis* Schmidt and Whittaker, 1975**

**Type hosts and type locality:** *Eleutherodactylus coqui* (YNQ)

**Host family:** Eleutherodactylidae

**Distribution:** Caribbean (Puerto Rico)

**References:** Schmidt and Whittaker (1975) and Ben Slimane et al. (1996a)

**Genus *Schulzia* Travassos, 1937**

***Schulzia chiribita* Durette-Desset, Florindez and Morales, 2000**

**Type hosts and type locality:** *Leptodactylus rhodonotus* (Günther) (YUL)

**Host family:** Leptodactylidae

**Distribution:** Neotropical (Peru)

**References:** Durette-Desset et al. (2000)

***Schulzia ptychoglossi* Bursey, Goldberg and Telford Jr., 2006**

**Type hosts and type locality:** *Alopoglossus festae* (Peracca) (=*Ptychoglossus festae*) (CRM)

**Host family:** Alopoglossidae

**Distribution:** Panamanian (Panama)

**References:** Bursey et al. (2006a)

***Schulzia travassosi* Durette-Desset, Baker and Vaucher, 1985**

**Type hosts and type locality:** *Rhinella crucifer* (= *Bufo crucifer*) (ANG)

**Hosts and locality records:** *Haddadus binotatus* (Spix) (ANG), *Ischnocnema guentheri* (Steindachner) (=*Hylodes guntheri, Eleutherodactylus guentheri*) (ANG), *Leptodactylus bufonius* (EES, INJ, LOM, TAC), *Leptodactylus latrans* (ANG), *Proceratophrys appendiculata* (Günther) (VDR), *Rhinella granulosa* (Spix) (= *Bufo granulosus*) (EES), *Rhinella icterica* (= *Chaunus ictericus*) (TRB), *Rhinella major* (CON, LOM), *Thoropa miliaris* (Spix) (=*Hylodes miliaris*) (CVD) and *Xenodon merremii* (Wagler) (= *Rhadinea merremii*).

**Host family:** Bufonidae, Leptodactylidae and Odontophrynidae

**Distribution:** Neotropical (Brazil, Paraguay, Argentina)

**References:** Travassos (1925), Travassos (1937), Durette-Desset et al. (1985), Boquimpani-Freitas et al. (2001), Bursey et al. (2006a), Lux Hoppe et al. (2008), González and Hamann (2015b), Hamann and González (2015), González et al. (2021).

**Remarks:** In 1866, Schneider described *Strongylus subventricosus*. This species was later transferred to the genus *Oswaldocruzia* by Travassos (1917) and mentioned again in Travassos (1921), though without clear justification. Travassos (1925) redescribed the species based on new material he collected. In 1937, Travassos established the genus *Schulzia*, designating *O. subventricosa* as its type species.

Durette-Desset et al. (1985) observed that materials from Schneider (1866) and Travassos (1925) allocated to *Schulzia subventricosa* represent distinct taxa. Since the species description provided by Travassos corresponded to his material only, the authors reclassified Schneider‘s specimens into the genus *Macielia* and introduced the name *Schulzia travassosi* for the material described 1925.

Consequently, *Macielia subventricosa* (Schneider) corresponds to *Strongylus subventricosus* Schneider and *Oswaldocruzia subventricosa* as defined by Travassos (1917, 1921). While *Schulzia travassosi* is equivalent to *O. subventricosa,* as defined by Travassos (1925).

***Schulzia usu* Lent and Portes Santos, 1989**

**Type hosts and type locality:** *Atelopus oxyrhynchus* Boulenger (MER)

**Host family:** Bufonidae

**Distribution:** Neotropical (Venezuela)

**References:** Lent and Portes Santos (1989)

**Genus *Typhlopsia* Baruš and Otero, 1978**

***Typhlopsia kratochvilli* Baruš and Coy Otero, 1978**

**Type hosts and type locality:** *Typhlops lumbricalis* (Linnaeus) (STC)

**Host family:** Typhlopidae

**Distribution:** Caribbean (Cuba)

**References:** Baruš and Coy Otero (1978) and Ben Slimane et al. (1996a)

**Figure 1.**
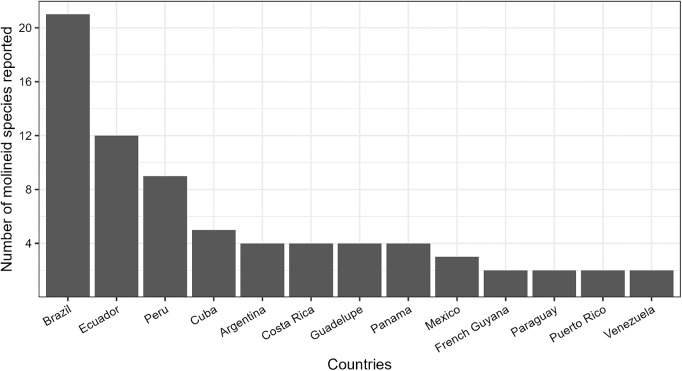
Countries with the highest number of molineid species recorded in this study. Countries are listed in descending order based on species count. Countries with only one recorded species are not shown.

In the Neotropical region, 36 molineid species have been recorded, of which 33 are endemic. In comparison, the Caribbean region has 11 species (all endemic), while Panamanian region has 9 species, with 6 being endemic. Among the 24 countries recorded, Brazil has the highest number of molineid species, totalling 21 species. It is followed by Ecuador with 12 species, Peru with 9 species and Cuba with 5 species. Other countries have fewer species registered: Argentina, Costa Rica, Guadeloupe and Panama have 4 species each; Mexico have 3 species, French Guiana, Paraguay, Puerto Rico and Venezuela have 2 species each. Finally, Bahamas, Bonaire, Chile, Dominica, Dominican Republic, Guyana, Haiti, Nicaragua, Saint Vincent and the Grenadines, Trinidad and Tobago and Uruguay all have 1 species each (see Figure 1).

**Figure 2.**
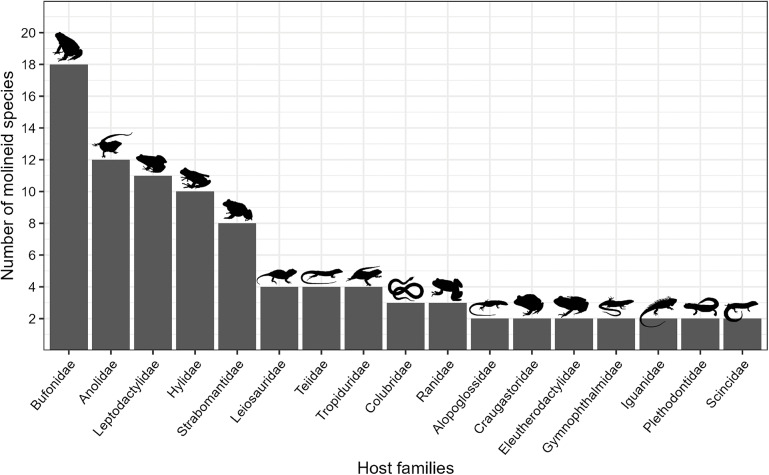
Number of molineid species per host family. Host families are ordered in descending order based on their number of species recorded. Families that have only one helminth species are not shown.

Among the 31 host families reported, 17 were parasitized by more than 1 species of molineid parasites. The families with higher molineid species identified were as follows: Bufonidae with 18 species, Anolidae with 12 species, Leptodactylidae with 11 species, Hylidae with 10 species and Strabomantidae with 8 species. The families Leiosauridae (Squamata), Teiidae (Squamata), Tropiduridae (Squamata) each had 4 species, while Colubridae (Squamata) and Ranidae with 3 species each. Additionally, the families Alopoglossidae, Craugastoridae, Eleutherodactulidae, Gymnophthalmidae, Iguanidae, Plethodontidae and Scincidae were represented by 2 species each. The remaining 14 host families had only 1 helminth species each, including Alsodidae, Batrachylidae, Brachycephalidae, Ceratophryidae, Cycloramphidae, Dendrobatidae, Gekkonidae, Hemiphractidae, Leiocephalidae, Microhylidae, Odontophrynidae, Phrynosomatidae, Tropidophiidae and Typhlopidae (Figure 2).

*Oswaldocruzia anolisi* and *O. subauricularis* were the most common molineid species overall. *Oswaldocruzia anolisi* was reported in 22 species from 6 families: Anolidae, Colubridae, Iguanidae, Leiocephalidae, Teiidae and Tropidophiidae). Similarly, *O. subauricularis* also was reported in 22 species of hosts from 6 families (Bufonidae, Ceratophryidae, Hylidae, Leiosauridae, Leptodactylidae and Ranidae) from Argentina, Brazil and Costa Rica.

Following these 2 species, *O. costaricensis* was reported in 18 species from 7 host families, *O. lenteixeirai* in 17 species from 4 families. Other species included *O. mazzai* (15 species from 6 families), *S. travassosi* (11 species from 7 families), *O. lopesi* (11 species from 6 families), *O. vitti* (10 species from 5 families), *O. proencai* (8 species from 3 families). Additionally, *O. nicaraguensis* was reported in 7 species from 3 families, *O. albareti* in 6 species from 3 families, *O. barusi* in 5 species from 1 family, *O. chabaudi* in 5 species from 1 family and *O. neghmei* in 5 species from 2 families.

**Figure 3.**
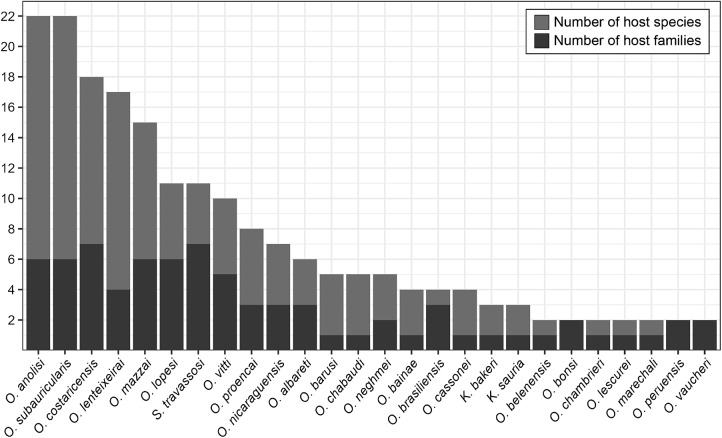
The most common species of molineid nematodes and their range of recorded hosts. Species are organized in descending order based on the number of host species (light grey), compared to the number of host families in which they occur (dark grey).

We should also highlight that *O. bainae* was found in 4 host species from 1 family, *O. brasiliensis* in 4 species from 3 families, *O. cassonei* in 4 species from 1 family. Lastly, *K. bakeri* and *K. sauria* each appeared in 3 species from 1 family. *Oswaldocruzia belenensis, O. chambrieri, O. marechali* and *O. lescurei* were found in 2 species from 1 family each, while *O. bonsi, O. peruensis* and *O. vaucheri* in 2 species from 2 families each (Figure 3). All 27 remaining taxa were reported for a single host species.

## Discussion

### Biogeographical division and species distribution

Ben Slimane et al. (1996a) classified the species of the genus *Oswaldocruzia* into 5 biogeographical groups based on differences in the spicular morphology, it aligns with the biogeographic division proposed by Sclater (1858) and adopted by Wallace (1876). In this division the Neotropical region encompasses the tropical and subtropical areas of the Americas, extending from southern Mexico to the southern tip of South America, including Central America and the Caribbean islands.

However, currently, 2 biogeographical realms proposed by Holt et al. (2013) that are equivalent to the former Neotropical realm, have been accepted. The Panamanian realm extends from Mexico to Panama, while the Neotropical realm, as it stands today, includes only the countries of South America. Thus, in the present study, we adopt the biogeographic distribution proposed by Holt et al. (2013).

Our results indicate that all 11 Caribbean species are endemic to that region. Only *O. bainae, O. lescurei* and *O. subauricularis* were found in more than 1 geographical region, specifically shared distribution among Panamanian and Neotropical regions. These findings provide evidence that molineid species from these regions represent distinct groups, particularly for the Caribbean species with a very particular morphology of spicules, as also observed by Ben Slimane et al. (1996a).

Brazil has the highest number of molineid species among the countries studied. This can be attributed to several factors, one of which is the fact that Brazil has the largest territorial area. This area encompasses diverse biomes and a rich diversity of hosts, which likely contributes to its rich parasite diversity, as noted by Campião et al. (2014). Also, other fact is the long-standing tradition of helminthology in South America, particularly in Brazil. Research on nematodes in Brazilian species dates back to 1648, with significant contributions from 19th-century expeditions of European researchers such as Rudolphi, Diesing and Molin, as well as the 20th-century contributions of Travassos and other prominent helminthologists (Vicente et al. 1993). In last decades, new research groups have been contributing to our knowledge on nematode diversity, regarding its herpetofauna (Ávila and Silva, 2010; Campião et al. 2014; Müller et al. 2018; Morais et al. 2020; Araujo Filho et al. 2020; Benício et al. 2022; Macedo et al. 2023, Vieira et al. (2023)).

Other South American countries also have a distinguished history in helminthology, with numerous authors and research groups dedicating significant efforts to the study of nematode diversity in Argentina (González and Hamann, 2015a; Draghi et al. 2020), Cuba (Baruš and Coy Otero, 1978), Ecuador (Ben Slimane and Durette-Desset, 1996b), Venezuela (Lent and Portes Santos, 1989; Ben Slimane et al. 1996b) and Peru (Bursey et al. 2005; Guerrero, 2013; Gómez et al. 2020; Cuellar et al. 2022; Luque and Chero, 2022), among several others.

### Unconfirmed registers and invalid names

There are 2 unconfirmed reports of molineid species from South American amphibians. Walton (1935) reported *O. filiformis* in *Ceratophrys aurita* (= *Ceratophrys dorsata*) and in *Leptodactylus latrans* (= *Leptodactylus ocellatus*) from Brazil and *O. pipiens* in *Leptodactylus* sp. from South America. Those reports are the only reference for these molineid species for South American amphibians. Travassos (1937) discussed that these identifications were inaccurate, and Freitas and Lent (1938) also commented that the identifications cannot be confirmed since *O. filiformis* and *O. pipiens* have been reported exclusively across the North America in a wide range of amphibians and reptiles. Thus, this report should be analysed carefully during species comparisons, and we did not consider valid in this study.

Travassos (1925) reported *O. subauricularis* from *Leptodactylus latrans* (= *Leptodactylus ocellatus*) in Brazil. However, Travassos (1937) later stated that the record of *O. subauricularis* from *L. latrans* was incorrect. Thus, we did not consider this report here.

The taxonomic history of *Schulzia* is complex. Travassos (1937) proposed this genus to allocate *Strongylus subventricosus* from Schneider (1866), designating *Schulzia subventricosa* as a type-species of the genus. These nematodes were previously placed within *Oswaldocruzia* by Travassos (1917) and described by Travassos (1925) with material from different hosts that he named *O. subventricosus*. However, the author provided no reasons for separating it into a new genus.

In 1985, Durette-Desset et al., identified nematodes similar to those described by Travassos (1925) and proposed the name *Schulzia travassosi* for specimens found in Paraguay. However, they noticed that the description given by Travassos (1937) corresponded only to the material he had described in 1925 and not from Schneider (1866). Consequently, Durette-Desset et al. (1985) reallocated Schneider‘s specimens to the genus *Macielia* Travassos (Nematoda, Trichostrongylidae). They redefined *Schulzia* by proposing the name *Schulzia travassosi* for Paraguayan specimens and material from Travassos (1925) and designating them as a type-species of the genus and *Macielia subventricosa* for Schneider‘s specimens. Thus, the name *Schulzia subventricosa* is currently not considered valid and not presented in this work.

### Host–parasite associations and specificity

We observed that, until now, molineid nematodes have been mainly found in amphibian hosts (146 species), while in reptiles, a lower number have been reported (74 species). The most common strategies used by nematodes to infect their hosts are by oral route or by skin penetration (Anderson, 2000). Studies on development and life cycle were conducted only for *O. filiformis* (Hendrikx 1981; Hendrikx 1983, Hendrikx and Van Moppes, 1983; Griffin, 1988) and for *O. pipiens* (Baker, 1978) which demonstrated that their cycles are direct. The infection route in these molineid nematodes is unclear, but one possible explanation of our results could be related to the direct cycle of these nematodes, particularly for skin penetration. Reptiles are covered in overlapping epidermal scales, reducing the area exposed to infection, whereas amphibians have smooth skins covered with mucus secretions that are more accessible to infective larvae.

Bufonids had more molineid species richness than all other amphibians’ host families. Also, for *R. marina* we observed the highest number of molineid species (11 species), followed by *R. margaritifera* (6 species). Willkens et al. (2021) proposed that the habitat use of the host (primarily terrestrial or predominantly arboreal) could account for the observed species diversity in certain amphibian families like Bufonidae. These terrestrial amphibians and their increased mobility compared to arboreal species makes contact with a higher diversity of parasites more likely (Poulin, 2007). Furthermore, several studies on helminth diversity have consistently demonstrated a higher functional diversity of parasite communities in terrestrial hosts (Campião et al. 2014; Hamann and González, 2015; Gómez et al. 2020; Euclydes et al. 2021). In contrast, considering that Bufonids are relatively common in these regions, and comparatively easier to collect and study, the apparent richness may partly reflect an artefact of rather different sampling effort.

Poulin (1997) suggested that host body size is a good predictor of parasite richness, as larger hosts may offer more habitats, ecological niches and resources than smaller ones. Similarly, Poulin (2007) proposed that larger hosts might harbour more parasites due to their greater surface area, higher food intake and the fact that they are typically older, providing more time for parasite accumulation. Hamann et al. (2006) and Campião et al. (2015) reported positive correlations between host body size and parasite richness in Neotropical amphibians, which likely explains why *Rhinella marina* exhibited the highest number of parasite species in the present study. However, Cardoso et al. (2021) found no significant influence of host body size on the composition of helminth taxa, suggesting that this pattern may not be consistent across all amphibian hosts.

The family Anolidae, represented by hosts of the genus *Anolis*, showed the second higher molineid species richness among all host families and the highest among reptiles, of which *Anolis marmoratus* presented the highest number of molineid species (4 species). This is the most abundant genus of lizards, and most species of this genus are arboreal or semi-arboreal (Uetz et al. 2025). Still, there are also terrestrial and semiaquatic species, of which the Caribbean species present particular ectomorphs that inhabit different niches (Losos, 1992) and might explain the richness observed for this family of reptiles.

Disregarding species reported only once and from a single host species, some nematodes occurred exclusively in a particular host family. For instance, *K. bakeri* was reported from 3 species of Hylidae (all from the genus *Boana*), *K. sauria* from 3 species of Teiidae (from the genera *Cnemidophorus* and *Kentropyx*), *O. chabaudi* from 6 different species of Hylidae (from the genera *Boana* and *Osteocephalus*), *O. lescurei* from 3 species of Bufonidae and *O. barusi* from 5 species of Bufonidae (all from the genus *Peltophryne*). These nematodes occurred in hosts with overlapping distributions and similar niches. Thus, this result might also reflect an association of these species with different habitats of their hosts, as Willkens et al. (2021) hypothesized. However, the authors also discussed a host-parasite cophylogeny hypothesis, which considers potential cospeciation events among hosts and their parasites and could partially explain our results.

Notably, other molineid species did not exhibit a specific association with a single host species or family. For instance, the most common species, *O. subauricularis* was found in 22 species of amphibian and reptilian hosts (Bufonidae, Ceratophryidae, Hylidae, Leiosauridae, Leptodactylidae, Ranidae and Strabomantidae) from Argentina, Brazil and Costa Rica. Similarly, *O. anolisi, O. costaricensis, O. lenteixeirai, O. mazzai, O. lopesi, O. vitti, O. proencai* and *O. nicaraguensis* also showed a broad host range. The occurrence of molineids infecting hosts from different families with different habitat uses has been reported previously (Bursey and Goldberg, 2011; Willkens et al. 2016). Also, we highlight that most species are poorly studied, and details regarding their distribution and host range are yet unknown.

Compiling and maintaining a comprehensive species list within the field of helminthology are indispensable for scientific research and advancements in understanding parasitic diversity. These species lists provide a systematic and organized record of all known helminth species, categorizing them based on taxonomic classifications, geographical distributions and host associations. Such information is crucial for researchers investigating helminths’ ecology, epidemiology and evolution.

In conclusion, our study and the checklist provided here, offers a comprehensive overview of nematodes of the family Molineidae, summarize details regarding their distribution and host range, and provide basis for further studies on this group. The taxonomic history of these Molineid genera and their species is complex, studies integrating morphological and molecular data are still required to achieve a broader understanding of their biology and to confirm their phylogenetic relationships and historical records. We also endorse that their diversity might still be underestimated, and new species and records from different localities and hosts are awaiting discovery.

## Supporting information

10.1017/S0031182025101364.sm001 Willkens et al. supplementary material Willkens et al. supplementary material

## Supplementary material

The Supplementary Material for this paper can be found at https://doi.org/10.1017/S0031182025101364.
