# Supplementary material for: Molineid nematodes of amphibians and reptiles: A checklist of Caribbean, Panamanian, and Neotropical species and notes on their biology and host associations
Source: Parasitology. 2025 Dec 12;153(2):169–85. doi: 10.1017/S0031182025101364 (PMC13215752; doi:10.1017/S0031182025101364)
Supplement: Willkens et al. supplementary material [file S0031182025101364sup001.docx]

| Species | Parasite |
| --- | --- |
| **Class Amphibia** |  |
| **Order Anura** |  |
| **Family Alsodidae** |  |
| *Alsodes vittatus* | *Oswaldocruzia neghmei* |
| *Eupsophus migueli* | *Oswaldocruzia neghmei* |
| *Eupsophus roseus* | *Oswaldocruzia neghmei* |
| *Eupsophus vertebralis* | *Oswaldocruzia neghmei* |
| **Family Batrachylidae** |  |
| *Hylorina sylvatica* | *Oswaldocruzia neghmei* |
| **Family Brachycephalidae** |  |
| *Ischnocnema guentheri* | *Schulzia travassosi* |
| **Family Bufonidae** |  |
| *Amazophrynella bokermanni* | *Oswaldocruzia chambrieri* |
| *Atelopus oxyrhynchus* | *Schulzia usu,* |
| *Incilius coccifer* | *Oswaldocruzia costaricensis* |
| *Incilius luetkenii* | *Oswaldocruzia costaricensis* |
| *Incilius marmoreus* | *Oswaldocruzia lescurei* |
|  | *Oswaldocruzia subauricularis* |
| *Peltophryne dunni* | *Oswaldocruzia barusi* |
| *Peltophryne empusa* | *Oswaldocruzia barusi* |
| *Peltophryne fustiger* | *Oswaldocruzia barusi* |
| *Peltophryne gundlachi* | *Oswaldocruzia barusi* |
| *Peltophryne taladai* | *Oswaldocruzia barusi* |
| *Rhaebo glaberrimus* | *Oswaldocruzia lopesi* |
| *Rhaebo haematiticus* | *Oswaldocruzia costaricensis* |
| *Rhinella arenarum* | *Oswaldocruzia proencai* |
| *Rhinella crucifer* | *Oswaldocruzia subauricularis* |
|  | *Schulzia travassosi* |
| *Rhinella diptycha* | *Oswaldocruzia mazzai* |
|  | *Oswaldocruzia proencai* |
|  | *Oswaldocruzia subauricularis* |
| *Rhinella dorbignyi* | *Oswaldocruzia subauricularis* |
| *Rhinella granulosa* | *Schulzia travassosi* |
| *Rhinella horribilis* | *Oswaldocruzia subauricularis* |
| *Rhinella icterica* | *Oswaldocruzia lopesi* |
|  | *Oswaldocruzia mazzai* |
|  | *Oswaldocruzia subauricularis* |
|  | *Schulzia travassosi* |
| *Rhinella major* | *Oswaldocruzia mazzai* |
|  | *Schulzia travassosi* |
| *Rhinella margaritifera* | *Oswaldocruzia albareti* |
|  | *Oswaldocruzia belenensis* |
|  | *Oswaldocruzia chambrieri* |
|  | *Oswaldocruzia lescurei* |
|  | *Oswaldocruzia lopesi* |
|  | *Oswaldocruzia mazzai* |
|  | *Oswaldocruzia proencai* |
| *Rhinella marina* | *Oswaldocruzia albareti* |
|  | *Oswaldocruzia belenensis* |
|  | *Oswaldocruzia costaricensis* |
|  | *Oswaldocruzia lamotheargumedoi* |
|  | *Oswaldocruzia lopesi* |
|  | *Oswaldocruzia manuensis* |
|  | *Oswaldocruzia mazzai* |
|  | *Oswaldocruzia subauricularis* |
|  | *Oswaldocruzia taranchoni* |
|  | *Oswaldocruzia urubambaensis* |
|  | *Oswaldocruzia venezuelensis* |
| *Rhinella ornata* | *Oswaldocruzia subauricularis* |
| *Rhinella* sp. | *Oswaldocruzia dlouhyi* |
|  | *Oswaldocruzia subauricularis* |
| **Family Ceratophryidae** |  |
| *Ceratophrys cornuta* | *Oswaldocruzia subauricularis* |
| **Family Craugastoridae** |  |
| *Craugastor fitzingeri* | *Oswaldocruzia costaricensis* |
| *Craugastor gollmeri* | *Oswaldocruzia costaricensis* |
| *Craugastor ranoides* | *Oswaldocruzia costaricensis* |
| *Craugastor taurus* | *Oswaldocruzia costaricensis* |
| *Haddadus binotatus* | *Schulzia travassosi* |
| **Family Cycloramphidae** |  |
| *Thoropa miliaris* | *Schulzia travassosi* |
| **Family Dendrobatidae** |  |
| *Ameerega picta* | *Oswaldocruzia lopesi* |
| **Family Eleutherodactylidae** |  |
| *Eleutherodactylus atkinsi* | *Oswaldocruzia lenteixeirai* |
| *Eleutherodactylus coqui* | *Oswaldocruzia lenteixeirai* |
|  | *Poekilostrongylus puertoricensis* |
| *Eleutherodactylus cuneatus* | *Oswaldocruzia lenteixeirai* |
| *Eleutherodactylus dimidiatus* | *Oswaldocruzia lenteixeirai* |
| *Eleutherodactylus goini* | *Oswaldocruzia lenteixeirai* |
| *Eleutherodactylus greyi* | *Oswaldocruzia lenteixeirai* |
| *Eleutherodactylus klinikowskii* | *Oswaldocruzia lenteixeirai* |
| *Eleutherodactylus pinarensis* | *Oswaldocruzia lenteixeirai* |
| *Eleutherodactylus planirostris* | *Oswaldocruzia lenteixeirai* |
| *Eleutherodactylus portoricensis* | *Oswaldocruzia lenteixeirai* |
| *Eleutherodactylus zeus* | *Oswaldocruzia lenteixeirai* |
| *Eleutherodactylus zugi* | *Oswaldocruzia lenteixeirai* |
| **Family Hemiphractidae** |  |
| *Gastrotheca peruana* | *Oswaldocruzia proencai* |
| **Family Hylidae** |  |
| *Agalychnis callidryas* | *Oswaldocruzia costaricensis* |
| *Boana boans* | *Kentropyxia bakeri* |
|  | *Oswaldocruzia chabaudi* |
| *Boana calcarata* | *Oswaldocruzia albareti* |
| *Boana faber* | *Oswaldocruzia subauricularis* |
| *Boana fasciata* | *Oswaldocruzia albareti* |
|  | *Oswaldocruzia chabaudi* |
|  | *Oswaldocruzia lopesi* |
| *Boana geographica* | *Kentropyxia bakeri* |
|  | *Oswaldocruzia albareti* |
|  | *Oswaldocruzia chabaudi* |
| *Boana raniceps* | *Oswaldocruzia mazzai* |
| *Boana wavrini* | *Kentropyxia bakeri* |
|  | *Oswaldocruzia chabaudi* |
| *Itapotihyla langsdorffiii* | *Oswaldocruzia chabaudi* |
| *Osteocephalus cabrerai* | *Oswaldocruzia chabaudi* |
| *Osteocephalus taurinus* | *Kentropyxia hylae* |
| *Osteopilus septentrionalis* | *Oswaldocruzia lenteixeirai* |
|  | *Oswaldocruzia moraveci* |
| *Phyllomedusa burmeisteri* | *Oswaldocruzia subauricularis* |
| *Scinax elaeochroa* | *Oswaldocruzia costaricensis* |
| *Smilisca phaeota* | *Oswaldocruzia costaricensis* |
| *Trachycephalus coriaceus* | *Oswaldocruzia lopesi* |
| *Trachycephalus mesophaeus* | *Oswaldocruzia subauricularis* |
| **Family Leptodactylidae** |  |
| *Leptodactylus bolivianus* | *Oswaldocruzia lopesi* |
| *Leptodactylus bufonius* | *Oswaldocruzia mazzai* |
|  | *Oswaldocruzia proencai* |
|  | *Schulzia travassosi* |
| *Leptodactylus fuscus* | *Oswaldocruzia mazzai* |
|  | *Oswaldocruzia proencai* |
|  | *Oswaldocruzia vaucheri* |
| *Leptodactylus latrans* | *Oswaldocruzia lopesi* |
|  | *Oswaldocruzia mazzai* |
|  | *Oswaldocruzia proencai* |
|  | *Oswaldocruzia subauricularis* |
|  | *Schulzia travassosi* |
| *Leptodactylus macrosternum* | *Oswaldocruzia franciscoensis* |
| *Leptodactylus melanonotus* | *Oswaldocruzia subauricularis* |
| *Leptodactylus mystaceus* | *Oswaldocruzia mazzai* |
| *Leptodactylus paraensis* | *Oswaldocruzia lanfrediae* |
| *Leptodactylus pentadactylus* | *Oswaldocruzia albareti* |
|  | *Oswaldocruzia mazzai* |
|  | *Oswaldocruzia petterae* |
|  | *Oswaldocruzia subauricularis* |
| *Leptodactylus pustulatus* | *Oswaldocruzia mazzai* |
|  | *Oswaldocruzia proencai* |
| *Leptodactylus rhodonotus* | *Schulzia chiribita* |
| *Physalaemus olfersii* | *Oswaldocruzia subauricularis* |
| **Family Microhylidae** |  |
| *Hamptophryne boliviana* | *Oswaldocruzia lopesi* |
| **Family Odontophrynidae** |  |
| *Proceratophrys appendiculata* | *Schulzia travassosi* |
| **Family Ranidae** |  |
| *Aquarana catesbeiana* | *Oswaldocruzia lenteixeirai* |
| *Lithobates brownorum* | *Oswaldocruzia subauricularis* |
| *Lithobates forreri* | *Oswaldocruzia costaricensis* |
|  | *Oswaldocruzia subauricularis* |
| *Lithobates sp.* | *Oswaldocruzia subauricularis* |
| *Lithobates taylori* | *Oswaldocruzia costaricensis* |
| *Lithobates vaillanti* | *Oswaldocruzia subauricularis* |
| *Lithobates warszewitschii* | *Oswaldocruzia costaricensis* |
| **Family Strabomantidae** |  |
| *Oreobates quixensis* | *Oswaldocruzia bonsi* |
|  | *Oswaldocruzia vaucheri* |
| *Pristimantis altamazonicus* | *Oswaldocruzia cassonei* |
|  | *Oswaldocruzia mazzai* |
|  | *Oswaldocruzia tcheprakovae* |
| *Pristimantis conspicillatus* | *Oswaldocruzia cassonei* |
| *Pristimantis diadematus* | *Oswaldocruzia cassonei* |
| *Pristimantis fenestratus* | *Oswaldocruzia lopesi* |
| *Pristimantis lanthanites* | *Oswaldocruzia cassonei* |
| *Pristimantis shrevei* | *Oswaldocruzia brevispicula* |
| *Pristimantis variabilis* | *Oswaldocruzia touzeti* |
| **Order Caudata** |  |
| **Family Plethodontidae** |  |
| *Bolitoglossa equatoriana* | *Oswaldocruzia bonsi* |
|  | *Oswaldocruzia cartagoensis* |
| **Class Reptilia** |  |
| **Order Squamata** |  |
| **Family Alopoglossidae** |  |
| *Alopoglossus angulatus* | *Oswaldocruzia vitti* |
| *Alopoglossus atriventris* | *Oswaldocruzia vitti* |
| *Alopoglossus festae* | *Schulzia ptychoglossi* |
| **Family Anolidae** |  |
| *Anolis allisoni* | *Oswaldocruzia anolisi* |
| *Anolis allogus* | *Oswaldocruzia anolisi* |
| *Anolis aquaticus* | *Oswaldocruzia costaricensis* |
| *Anolis armouri* | *Oswaldocruzia lenteixeirai* |
| *Anolis bahorucoensis* | *Oswaldocruzia lenteixeirai* |
| *Anolis baracoae* | *Oswaldocruzia anolisi* |
| *Anolis bartschi* | *Oswaldocruzia anolisi* |
| *Anolis biporcatus* | *Oswaldocruzia bainae* |
| *Anolis biporcatus* | *Oswaldocruzia nicaraguensis* |
| *Anolis bonairensis* | *Oswaldocruzia lenteixeirai* |
| *Anolis brasiliensis* | *Oswaldocruzia mazzai* |
| *Anolis bremeri* | *Oswaldocruzia anolisi* |
| *Anolis capito* | *Oswaldocruzia nicaraguensis* |
| *Anolis chrysolepis* | *Oswaldocruzia bainae* |
| *Anolis equestris* | *Oswaldocruzia anolisi* |
| *Anolis fuscoauratus* | *Oswaldocruzia bainae* |
|  | *Oswaldocruzia vitti* |
| *Anolis homolechis* | *Oswaldocruzia anolisi* |
| *Anolis humilis* | *Oswaldocruzia nicaraguensis* |
| *Anolis limifrons* | *Oswaldocruzia nicaraguensis* |
| *Anolis lionotus* | *Oswaldocruzia costaricensis* |
|  | *Oswaldocruzia nicaraguensis* |
| *Anolis loysiana* | *Oswaldocruzia anolisi* |
| *Anolis lucius* | *Oswaldocruzia anolisi* |
| *Anolis luteogularis* | *Oswaldocruzia anolisi* |
| *Anolis marmoratus* | *Oswaldocruzia dorsarmata* |
|  | *Oswaldocruzia jeanbarti* |
|  | *Oswaldocruzia marechali* |
|  | *Oswaldocruzia mauleoni* |
| *Anolis oculatus* | *Oswaldocruzia marechali* |
| *Anolis porcus* | *Oswaldocruzia anolisi* |
| *Anolis punctatus* | *Oswaldocruzia peruensis* |
|  | *Oswaldocruzia vitti* |
| *Anolis quadriocellifer* | *Oswaldocruzia anolisi* |
| *Anolis sagrei* | *Oswaldocruzia anolisi* |
| **Family Colubridae** |  |
| *Caraiba andreae* | *Oswaldocruzia anolisi* |
| *Cubophis cantherigerus* | *Oswaldocruzia anolisi* |
| *Erythrolamprus miliaris* | *Oswaldocruzia brasiliensis* |
| *Palusophis bifossatus* | *Oswaldocruzia brasiliensis* |
| *Xenodon merremii* | *Schulzia travassosi* |
| **Family Gekkonidae** |  |
| *Hemidactylus mabouia* | *Oswaldocruzia brasiliensis* |
| **Family Gymnophthalmidae** |  |
| *Cercosaura eigenmanni* | *Oswaldocruzia vitti* |
| *Cercosaura ocellata* | *Oswaldocruzia vitti* |
| *Cercosaura oshaughnessyi* | *Oswaldocruzia vitti* |
| *Loxopholis rugiceps* | *Oswaldocruzia panamaensis* |
| **Family Iguanidae** |  |
| *Ctenosaura quinquecarinata* | *Oswaldocruzia costaricensis* |
| *Cyclura nubila* | *Oswaldocruzia anolisi* |
| **Family Leiocephalidae** |  |
| *Leiocephalus carinatus* | *Oswaldocruzia anolisi* |
| *Leiocephalus cubensis* | *Oswaldocruzia anolisi* |
| *Leiocephalus macropus* | *Oswaldocruzia anolisi* |
| *Leiocephalus stictigaster* | *Oswaldocruzia anolisi* |
| **Family Leiosauridae** |  |
| *Enyalius bilineatus* | *Oswaldocruzia benslimanei* |
| *Enyalius iheringii* | *Oswaldocruzia fredi* |
| *Enyalius perditus* | *Oswaldocruzia burseyi* |
|  | *Oswaldocruzia subauricularis* |
| **Family Phrynosomatidae** |  |
| *Sceloporus variabilis* | *Oswaldocruzia costaricensis* |
| **Family Scincidae** |  |
| *Copeoglossum nigropunctatum* | *Oswaldocruzia brasiliensis* |
| *Scincella cherriei* | *Oswaldocruzia nicaraguensis* |
| **Family Teiidae** |  |
| *Cnemidophorus gramivagus* | *Kentropyxia sauria,* |
|  | *Oswaldocruzia vitti* |
| *Holcosus festivus* | *Oswaldocruzia nicaraguensis* |
| *Kentropyx calcarata* | *Kentropyxia sauria* |
| *Kentropyx pelviceps* | *Kentropyxia sauria* |
| *Pholidoscelis auberi* | *Oswaldocruzia anolisi* |
| **Family Tropidophiidae** |  |
| *Tropidophis pardalis* | *Oswaldocruzia anolisi* |
| **Family Tropiduridae** |  |
| *Plica plica* | *Oswaldocruzia vitti* |
| *Plica umbra* | *Oswaldocruzia bainae,* |
|  | *Oswaldocruzia vitti* |
| *Stenocercus roseiventris* | *Oswaldocruzia peruensis* |
| *Tropidurus torquatus* | *Oswaldocruzia mazzai* |
| **Family Typhlopidae** |  |
| *Typhlops lumbricalis* | *Typhlopsia kratochvilli* |
